# Phenotypic and Functional Characterization of Lymphocytes from Different Age Groups of Bolivian Squirrel Monkeys (*Saimiri boliviensis boliviensis*)

**DOI:** 10.1371/journal.pone.0079836

**Published:** 2013-11-25

**Authors:** Pramod N. Nehete, Patrick W. Hanley, Bharti P. Nehete, Guojun Yang, Julio C. Ruiz, Lawrence Williams, Christian R. Abee, K. Jagannadha Sastry

**Affiliations:** 1 Department of Veterinary Sciences, The University of Texas MD Anderson Cancer Center, Bastrop, Texas, United States of America; 2 Department of Immunology, The University of Texas MD Anderson Cancer Center Houston, Texas, United States of America; University of Nebraska Medical Center, United States of America

## Abstract

Due to many physiological and genetic characteristic similarities to humans, squirrel monkeys provide an ideal animal model specifically for studying malaria, and transmissible spongiform encephalopathies (Creutzfeldt-Jacob disease). While squirrel monkeys three years and older are generally considered adult subjects suitable for use in medical research studies, little is known about the functional properties of lymphocytes in relation to the age of these animals, which could significantly impact the quality and quantity of innate and adaptive immune responses. In this study, we investigated differences in the phenotype and function of lymphocytes subsets of young (3–4 years), adult (8–10 years) and aged (16–19 years) squirrel monkeys. In general, animals in all three age groups exhibited comparable numbers of different lymphocyte subsets except for CD20+ B cells that were significantly lower in aged relative to young animals and T cells subsets expressing both CD4 and CD8 (double positive) were significantly higher in aged relative to young animals. With increasing age, phenotypic differences in central and effector memory T cells subsets were observed, that were more pronounced for the CD8+ T cells. Despite equal proportions of CD3+ T cells among the three age groups, responses of peripheral blood mononuclear cells to T cell mitogens PHA and Con A showed lower IFN-γ producing cells in the aged group than that in the young group. Furthermore, aged animals showed significantly higher plasma levels of inflammatory cytokines IL-6, IFN-γ, TNF-α, IL-10 and IL-12. These findings suggest that while the squirrel monkeys in general share phenotypic and functional similarities of lymphocyte subsets with humans in relation to age, specific differences exist in immune function of lymphocytes between young and old animals that could potentially impact experimental outcomes for which the measurement of immunologic endpoints are critical.

## Introduction

Due to phylogenetic closeness to humans, nonhuman primates often provide the best animal models for human infectious disease or infectious disease sequelae investigations. Squirrel monkeys harbor known endemic viruses that are analogues of opportunistic human viruses such as: the squirrel monkey polyomavirus (SMPyV), the squirrel monkey cytomegalovirus (SM-CMV), Saimiriine Herpesvirus-1 (SaHV-1), Saimiriine Herpesvirus-2 (SaHV-2) and Saimiriine Herpesvirus-3 (SaHV-3) [1]–[4]. Furthermore, squirrel monkeys are an important animal model for malaria vaccine development [5], [6] due to their susceptibility to some of the same strains that cause disease in humans. In addition, the squirrel monkey is recognized as one of the most susceptible nonhuman primate species to experimental transmission of Creutzfeldt-Jakob disease (CJD) and other transmissible spongiform encephalopathies [7].

Age-related changes in immune function and their associated incidence of infections, cancer, autoimmune and immune complex diseases have been well studied in humans, rhesus macaques and mice [8]–[12]. The exact mechanisms underlying these alterations are poorly understood. For example, it is not known if the age-associated impairment of the peripheral mononuclear cells (PBMC) response to T cell mitogens is secondary to a decrease in circulating T cells [13], to reduced T-cell proliferative potential, to increased suppressor T-cell activity or to alterations of the cellular interactions involved in the proliferative response to mitogens [14]–[16]. Several contradictory reports in the literature ascribe the age-related influences on the numbers of circulating T lymphocytes [9]; changes in the frequency of memory CD4+T cells [10] changes in subsets and their functions [9], [14]; or phenotype shift from naïve to memory effector cells [17].

Even though the squirrel monkey has been used as an experimental human disease model for over a decade, very little has been published regarding the normal phenotype and function of their immune system. To the best of our knowledge, the only report describing the immune status in squirrel monkeys concentrates on lymphocyte surface antigen expression [18]. In the present study, we have analyzed for immunological characteristics of squirrel monkeys (*Saimiri boliviensis boliviensis*) in three different age groups for deciphering potential age-associated changes in lymphocyte populations and functions.

## Materials and Methods

### Monkeys, Care and Housing

Subject animals consisted of 30 female *Saimiri boliviensis boliviensis* randomly selected from the Squirrel monkey Breeding Research Resources (SMBRR) at the UT MD Anderson Cancer Center Michale E. Keeling Center for Comparative Medicine and Research. Social-breeding groups at the SMBRR consist of one male and 8–12 females with varying numbers of juveniles, housed in two connecting cages that are 4′ wide×6′ tall×14′ long.

### Ethics Statement

This research was conducted at the AAALAC-I accredited Michale E. Keeling Center for Comparative Medicine and Research, The UT MD Anderson Cancer Center, Bastrop, TX (Common Squirrel monkeys 03-09-02781). All animal experiments were carried out according to the provisions of the Animal Welfare Act, PHS Animal Welfare Policy, and the principles of the NIH Guide for the Care and Use of Laboratory Animals. All procedures were approved by the Institutional Animal Care and Use Committee at the UT MD Anderson Cancer Center.

### Diet

Animals have *ad libitum* access to New World Primate Diet (Purina #5040) and water. In addition, they are fed either a fresh fruit or vegetable daily. Specialty foods, such as seeds, peanuts, raisins, yogurt, cereals, frozen juice cups and peanut butter, are distributed daily to them as enrichment. At no time were the subjects ever food or water deprived. Subjects were also provided with destructible enrichment manipulanda and different travel/perching materials on a rotating basis to promote the occurrence of typical species behavior.

### Study Groups

The study population consisted of 30 female squirrel monkeys ranging from 3–19 years of age randomly selected and distributed between three groups ranging from a 3–4 years as young, 8–10 year as adult, and 16–19 as an aged group. Each group consists of 10 monkeys. Blood samples were collected from study animals at two time points four to six weeks apart. All subjects were considered healthy and in their normal social groups at the time they were sampled. Animals greater than 20 years of age were not included in this study due to age associated chronic diseases. Squirrel monkeys average life span is 20 years and maximum life expectancy of <25 years [19]. With an estimated maximum life-span of 122 years in humans [19]–[21], the rate of ageing in squirrel monkeys is roughly 3.4 times as fast. Thus, squirrel monkeys offer a distinct advantage over long-term human ageing research, but longitudinal studies in these primates require a major investment of time, resources, and effort.

### Clinical and Laboratory Assessment of Study Animals

Animals were observed and clinically evaluated daily by the clinical veterinarian as part of a routine colony maintenance protocol. Physical examinations occurred at sampling time points and as needed based on clinical observations. Blood samples were taken from a peripheral vein and analyzed for a complete blood count (Siemens Advia 120 Hematology Analyzer, Tarrytown, NY) and serum chemistry profile (Olympus AU400e® Chemistry Immuno Analyzer, Brea, CA). A manual white blood cell differential (including nucleated red blood cell count) was also performed.

### Blood Collection and PBMC Preparation

Blood sample (2–3 ml) was collected in EDTA coated collection tubes and immediately plasma was separated by centrifugation and stored at −80°C till further use. PBMCs were isolated by Ficoll-Hypaque density gradient separation as described previously [22], [23]. Erythrocytes were removed by osmotic lysis in ACK lysing buffer (Life Technologies, Grand Island, NY), and the remaining nucleated cells were washed twice with RPMI supplemented with 10% fetal bovine serum (FBS) and used for immune assay.

### Flow Cytometry

A series of commercially available human monoclonal antibodies were tested for cross reactivity to *Saimiri boliviensis* mononuclear cells using flow cytometric analysis. Cell-surface markers were determined using the following fluorescence labeled monoclonal antibodies specific to different lymphocytes subsets: CD3 (FITC, clone SP34-2, BD Pharmingen), CD4 (PE, clone L200, BD Pharmingen, San Jose, CA), and NK cells (CD16 PE clone 3G8, BD Pharmingen, San Jose, CA), and B cells (CD20+ APC clone L27, BD pharmingen, San Jose, CA) and CD8 (PE, clone B5, Invitrogen, Carlsbad, CA). Phenotypic characterization of lymphocytes in peripheral blood from the monkeys was performed by cell staining and flow-cytometric analysis as described previously [23], [24]. Briefly, 100 µl of EDTA-preserved whole blood from each sample was added to individual 12 mm×75 mm polystyrene test tubes (Falcon, Lincoln Park, NJ, USA) containing pre-added monoclonal antibodies against CD3, CD4 and CD8, and CD20 and incubated for 15 min at room temperature in the dark. After removing the red blood cells by incubating with FACS lysing solution (Becton Dickinson, USA), the mononuclear cells were washed twice with phosphate-buffered saline (PBS) and re-suspended in 2% formaldehyde. A separate tube of 100 µL of blood was used for staining with the combination of anti-CD3 (FITC, clone SP-34); and anti-CD16 (PE cline 3G8, BD Pharmingen) antibodies as described above. The stained cells were acquired with FACSCalibur (Becton Dickinson, CA, USA) equipped with a 488 nm argon ion laser and a 635 nm red diode laser. The CD3+ cells from lymphocytes that were gated on forward scatter versus side scatter dot plot were used to analyze for CD4+ and CD8+ lymphocyte subsets using Cell QuestPro software (Becton Dickinson, CA, USA). All antibodies used in this study are cross reactive to *Saimiri boliviensis* as reported in NIH Nonhuman Primate Reagent Resource core facility.

For the analyses of T cell memory subsets, EDTA-preserved whole blood was stained with the following fluorescence labeled monoclonal antibodies: anti-CD3 (FITC, clone SP34-2, BD Biosciences, San Jose, CA), anti-CD4 (PE, clone L200, BD Biosciences), anti-CD28 (PerCpCy5.5 BD Biosciences), and anti-CD95 (APC, clone DX2, BD Biosciences). One hundred microliters of blood were stained with antibodies for 15 min at the room temperature and red blood cells (RBC) were then lysed with 1× FACS lysing solution (BD Biosciences). After washing the cells twice with PBS containing 2% FBS, the cells were fixed with PBS containing 1% formaldehyde (Sigma, St. Louis, MO). Both compensation controls and fluorescence minus one (FMO) controls were utilized. Results were acquired on a FACSCaliber (BD Biosciences) and analysed using FlowJo software (Tree Star, Inc., Ashland, OR). In some experiments samples were also acquired on an LSRII flow cytometer (BD Biosciences, San Jose, CA) using live-dead fixable dead cells stain kit obtained from Invitrogen (Carlsbad, CA).

### Cytokine Multiplex Assays

Cytokines were measured in the plasma samples from EDTA preserved whole blood using nonhuman Primate Cytokine kit with IL-2, IL-4, IL-6, IL-10, IL-12/23(p40), IFN-γ and TNF-α from Millipore Corporation (Billerica, MA). There is 91.4%–98.1% homology between the nucleotide sequences of squirrel monkey cytokines genes and published sequences of equivalent human and nonhuman primate genes [25]. Plasma concentrations of cytokines were determined using the cytokine bead array (CBA) methodology according to the manufacturers’ protocols. Briefly, EDTA-preserved plasma samples were centrifuged (14,000×g for 5 minutes) and aliquots were frozen at −80°C until used. Prior to assay, once-thawed plasma samples were pre-cleared by centrifuging at 14,000×g for 5 minutes. The 96-well filter plate was blocked with assay buffer for 10 min at room temperature, washed, and 25 µl of standard or control samples were dispersed in appropriate wells. After adding 25 µl of beads to each well, plate was incubated on shaker overnight at 4°C. Next day, after washing two times with wash buffer, plate was incubated with detection antibody for 1 hr at room temperature and again incubated with 25 µl of Streptavidin-Phycoerythin for 30 min at room temperature. After washing two times with wash buffer, in each well 150 ul of sheath fluid was added and multianalyte profiling was performed on the Bio-Plex 200 system (Luminex X MAP technology). Calibration microspheres for classification and reporter readings as well as sheath fluid, assay and wash buffer were also purchased from Bio-Rad (Hercules, CA). Acquired fluorescence data were analyzed by the Bio-Plex manager 5.0 (from Bio-Rad, Hercules, CA). All steps of incubations were performed on a shaker. The minimum detectable concentration was calculated by the Multiplex Analyst immunoassay analysis Software from Millipore. The minimum detectable concentrations in pg/ml for the various cytokines are as follows: IL-2 (0.7), IL-4 (2.7), IL-6 (0.3), IL-10 (6.2), IL-12(P40) (1.2), IFN- γ (2.2), and TNF- α (2.1).

### *In Vitro* Mitogen Stimulation

The PBMC prepared from the blood samples by the standard ficoll-hypaque density-gradient centrifugation were used for various immunoassays. The proliferation of PBMC samples from the monkeys obtained during the study were determined by the standard [^3^H] thymidine incorporation as described before [22], [24]. Briefly, aliquots of the PBMC (10^5^/well) were seeded in triplicate wells of 96-well plates and stimulated for 6 days individually with the mitogens Concanavalin-A (Con A), phytohemagglutinin (PHA), lipopolysaccharide (LPS) and pokeweed mitogen (PWM) (each at 5 µg/ml final concentration) (Sigma. St Louis. MO). The culture medium without added mitogens served as negative control. After culture for 5 day at 37°C in 5% CO_2_, each well was pulsed for 18 h with 0.1 µCi of methyl-^3^H-thymidine (ICN. Irvine, CA). These mitogen concentrations, PBMC numbers and incubation times were found to be optimal conditions for stimulation of PBMC from healthy animals in our laboratory. The contents of the wells were then harvested onto glass fiber discs using a Skatron cell harvester (Skatron Laboratories, VA, and USA). The amount of radioactivity was determined in a Wallac Liquid Scintillation Counter (Wallac1409, Mustionkatu, Tarku, Finland). Mitogen responsiveness of each individual sample was expressed as net counts per minute (CPM) and calculated as: ΔCPM = (CPM with mitogen)−(CPM without mitogen).

### ELISPOT Assay for Detecting Antigen-specific IFN-γ Producing Cells

Freshly-isolated PBMC as described above, were stimulated with the mitogens PHA, Con A, LPS and PWM (each at 5 µg/ml final concentration) to determine the numbers of IFN-γ-producing cells by the Enzyme Linked Immuno Spot (ELISPOT) assay using the methodology reported earlier [22], [23]. Briefly, aliquots of PBMC (10^5^/well) were seeded in triplicate wells of 96-well plates (polyvinylidene difluoride backed plates, MAIP S 45, Millipore, Bedford, MA) pre-coated with the primary IFN-γ antibody and the lymphocyte were stimulated with the different mitogens. After incubation for 30–32 hr. at 37°C, the cells were removed and the wells were thoroughly washed with PBS and developed as per protocol provided by the manufacturer. Purple colored spots representing individual cells secreting IFN-γ were counted by an independent agency (Zellnet Consulting, New Jersey, NJ) using the KS-ELISPOT automatic system (Carl Zeiss, Inc. Thornwood, NY) for the quantitative analysis of the number of IFN-γ spot forming cells (SFC) for 10^5^ input PBMC. Responses were considered positive when the numbers of SFC with the test antigen were at least five and also were five above the background control values from cells cultured in the medium alone.

### Statistical Analysis

For statistical analysis, samples were grouped according to age of the animals from which samples were obtained. Comparison between groups of monkeys was done by one-way analysis of variance with the Kruskal-Wallis test and Gaussian approximation with Dunn’s multiple comparison tests. Only differences with a probability less than 0.05 were considered to be significant. All statistical analyses were conducted using GraphPad Prism® 5.00 (GraphPad Software, San Diego, California USA).

## Results

### Clinical Laboratory Assessment of Study Animals

Whole blood samples from animals in the three different age groups were subjected to comparative analyses of a panel of hematologic parameters like red blood cells (RBC), hemoglobin (HGB), hematocrit (HCT), mean corpuscular volume (MCV), mean corpuscular hemoglobin (MCH), mean corpuscular hemoglobin concentration (MCHC), and red blood cell distribution (RDW). As show in Table 1, we did not observe significant differences for any of the parameters between young, adult and aged animals.

**Table 1:** Comparison of Hematologic Parameters Between young, Adult and aged female.

Parameters	Young (Mean ± SD)	Adult (Mean ± SD)	Aged (Mean ± SD)
Segmented neutrophils (10^∧^3/ul)	3.24±2.05	3.06±1.27	3.60+2.62
Lymphocyte (10^∧^3/ul)	3.26±0.62	3.56+1.84	3.43+1.16
Monocyte (10^∧^3/ul)	0.32±0.29	0.28±0.21	0.23+0.23
Eosinophils(10^∧^3/ul)	0.17±0.13	0.168±0.1	0.17+0.11
WBC×10^∧^3/ul	6.94±2.4	7.1±2.59	7.42+2.33
RBC (×10^∧^6/ul)	6.74±0.26	6.9±0.29	6.78+0.46
HGB (g/dl)	13.28±0.66	12.89±0.39	13.33+0.69
HCT (%)	41.16±2.8	40.8±1.35	41.5+1.27
MCV (FL)	61.05±3.8	59.13±2.33	61.09+3.22
MCH (pg)	19.68±0.82	18.68±0.38	19.58+0.67
MCHC (g/dl)	32.31±1.59	31.61±0.82	32.15+1.26
RDW (%)	12.65±0.61	12.31±0.22	12.76+0.6
PLT (×10^∧^3/ul)	291.33±135.5	395.72±80.28	490.4+161.3
MPV (fL)	12.11±1.4	10.94±1.48	11.02+1.39

Abbreviation of tests: White Blood Cells (WBC), Red blood cells (RBC), Hemoglobin (Hgb), Hematocrit (HCT), Mean Corpuscular volume (MCV), Mean Corpuscular Hemoglobin (MCH), Mean Corpuscular Hemoglobin Concentration (MCHC), and Red Blood Cell Distribution (RDW), Platelet Counts (PLT), Mean platelet Volume (MPV).

### Phenotypic Characterization of Lymphocytes

**Figure 1:**
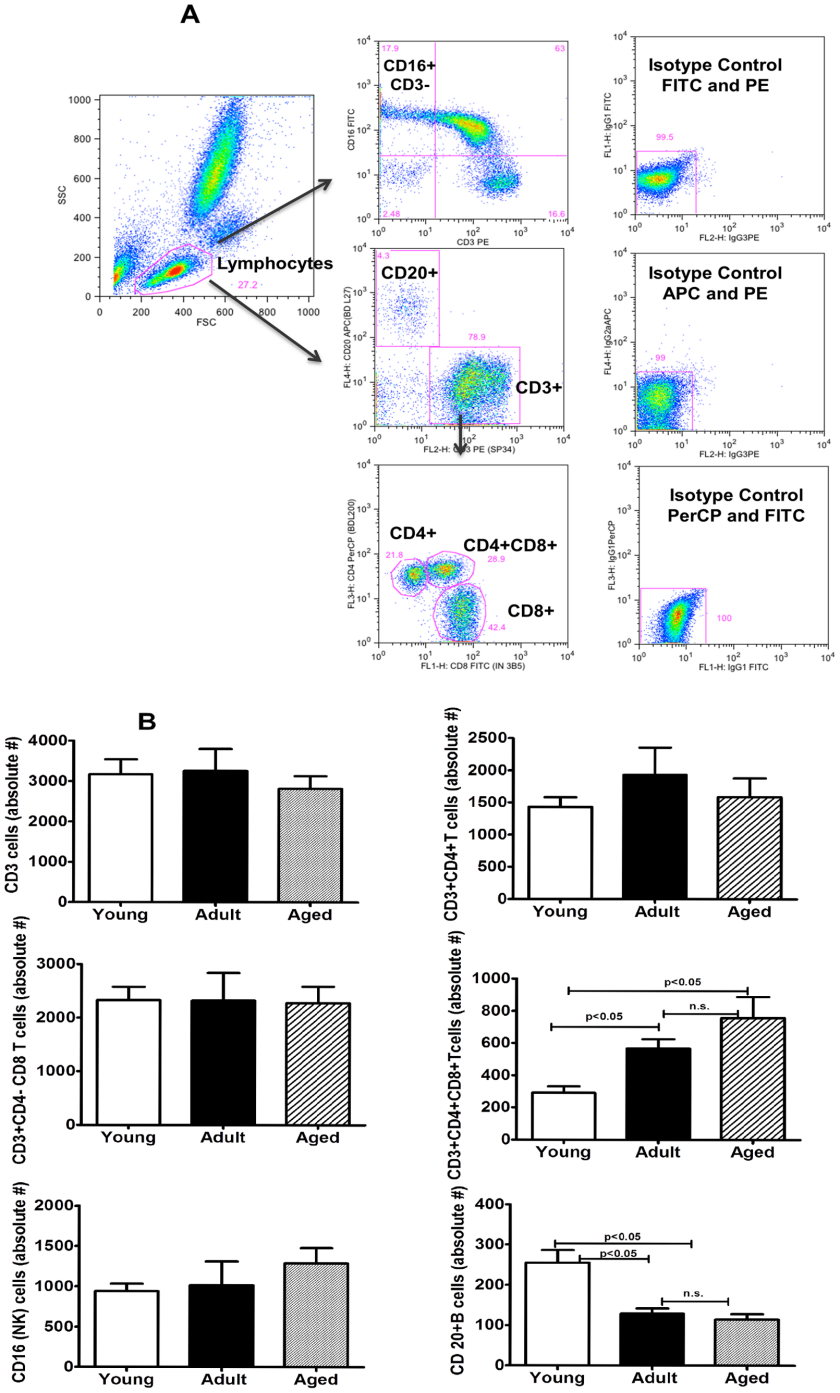
Phenotypic analyses of lymphocytes in young, adult and aged groups of female squirrel monkeys. (**A**) Gating scheme for phenotype analyses of the different cell markers in the peripheral blood from a representative animal. The lymphocytes were first gated based on forward scatter (FCS) versus side scatter (SSC) and then CD3+ T cells and CD20+ B cells were positively identified. Further analyses of CD3+ T cells show CD4+ T cells, CD8+ T cells and CD4+CD8+ double positive T cells. The specificity of staining for the different markers is ascertained based on isotype control antibody staining used for each pair of combination markers as shown. (**B**) Numbers of different lymphocyte populations in the three groups of animals: Aliquots of EDTA whole blood were stained with fluorescence labeled antibodies to the CD3+, CD4+, CD8+, CD20+ and CD16+ lymphocytes. Values on the Y-axis are the absolute number of Lymphocytes cells. The results shown are average of two separate experiments and the standard deviation values did not exceed 15% of the mean value. P values were considered at p<0.05.

**Figure 2:**
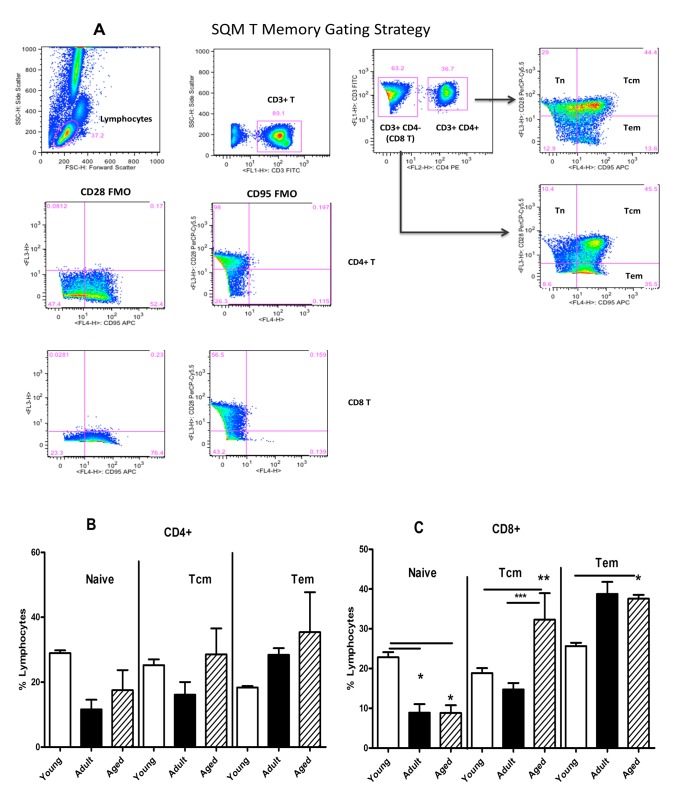
Phenotypic analyses of memory T cell subpopulations. (**A**) Gating scheme for the analyses of the different T cell subsets in the peripheral blood from a representative animal. The lymphocytes were first gated based on forward scatter (FCS) versus side scatter (SSC), and then live lymphocytes were identified based on SSC and live population (the later based on Aqua Live/Dead reagent (Invitrogen, Carlsbad, CA). The T cells were then positively identified by CD3 expression followed by the detection of the CD4+ CD8− (CD4+ T cells) and CD4− CD8+ (CD8+ T cells) populations within the CD3+ T cells. On the basis of CD28 and CD95 expression, the CD4+ and CD8+ T cells were further differentiated into naive (Tn CD28+ CD95−), central memory (Tcm CD28+ CD95+) and effector memory (Tem CD28− CD95+) subsets. The specificity of staining for the different markers is ascertained based on fluorescence minus one (FMO) controls shown and as described in the methods section. Blood samples from the three different age groups of squirrel monkeys were stained, and analysed for T cell subpopulations by flow cytometry as described in the methods section. Percentages of naïve (CD28+ CD95−), central memory (CD28+CD95+), and effector memory (CD28−CD95+) subsets of CD4 (**B**) and CD8 T cells (**C**) were compared between the three different groups. The results shown are average of 10 monkeys in each group and *P*<0.05 was considered statistically significant.

**Figure 3:**
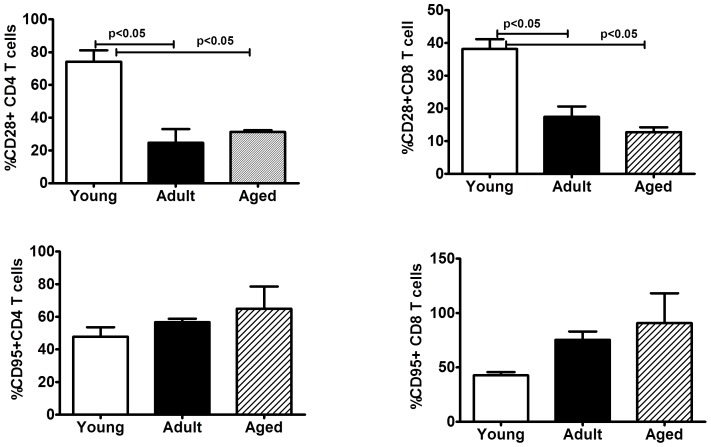
Phenotypic analyses of co-stimulatory (CD28) and apoptotic (CD95) markers on T-cell subsets. Percentages of CD28+ and (upper panel) and CD95+ populations (lower panel) within CD4+ and CD8+ T cells in the peripheral blood were compared in the three different age groups of squirrel monkeys. The results shown are average of 10 monkeys in each group and *P*<0.05 was considered statistically significant.

Peripheral blood samples from the monkeys in the three different age groups selected were analyzed by flow-cytometry for enumerating different lymphocyte subsets. A gating strategy for phenotypic analysis of T and B cells is shown in Fig. 1A. Age-related differences were not observed for lymphocytes that are CD3+, CD4+, CD8+, and CD16+NK cells, but significantly high numbers of CD4+CD8+ (double positive) T cells (p<0.05) were observed in the aged group of animals, relative to animals in the adult as well as young groups (Fig. 1B). On the other hand, B cells expressing the CD20 marker while being similar between animals in the adult and aged groups of monkeys were significantly lower relative to those in the young group. The different memory and naïve subsets of CD4+ and CD8+ T cells were enumerated by flow cytometry using the gating strategy shown in Fig. 2A. Among the three different age groups of the animals no significant differences were observed for the naïve or central and effector memory subsets of CD4+ T cells (Fig. 2B). However, as shown in Fig. 2C, within the CD8+ T cells subsets, significantly higher numbers of CD8+ central memory T cells were observed in aged monkeys relative to those in the young and adult groups (p<0.05). The CD8+ effector memory T cells in the adult and aged groups of animals were comparable, but were both significantly higher than those in the animals from the young group (Fig. 2C). We also analysed the expression of CD28 and CD95 individually and observed an age-associated reduction for CD28 and increase for CD95 on both CD4+ and CD8+ T cells, (Fig. 3).

### Functional Characterization of Lymphocytes

#### Proliferative responses

**Figure 4:**
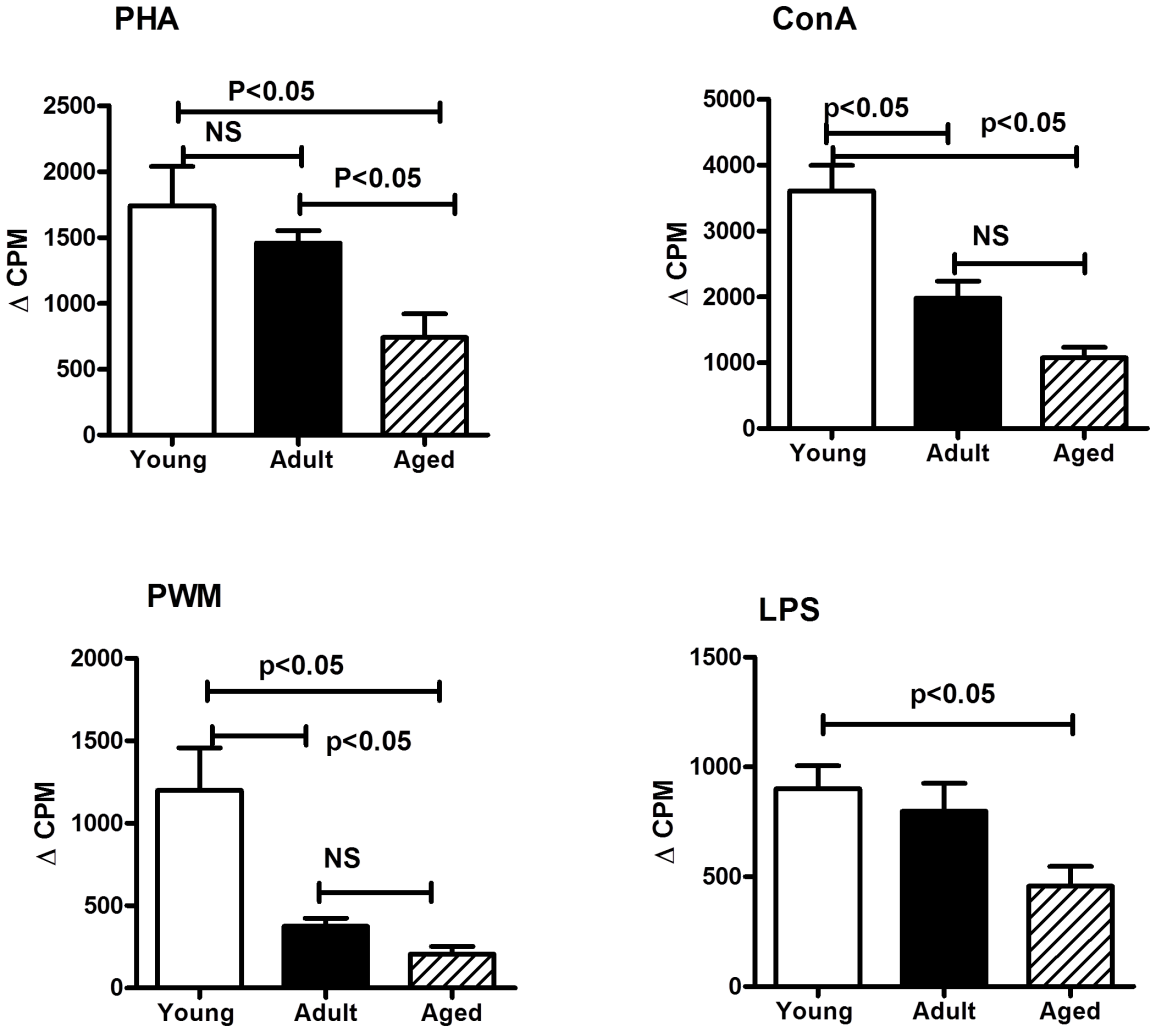
Proliferative response of PBMC to mitogens. PBMC isolated from the blood samples of the squirrel monkeys were used for determining proliferative response to different mitogens using the standard [3H] thymidine incorporation assay. The proliferation responses are expressed as delta (Δ) counts per minuets (CPM), representing increase in radioactivity incorporated in the presence of the mitogen over to that in medium control. The results shown are average of two separate experiments and the standard deviation values did not exceed 15% of the mean value. P values were considered at p<0.05.

The proliferation of PBMC samples from the monkeys were determined by the standard [3H] thymidine incorporation assay using the mitogens PHA, Con A, PWM and LPS and the results are shown in Fig. 4. Animals in the aged as well as adult groups showed significantly lower proliferative responses to Con A (p<0.05), PHA (p<0.05), PWM (p<0.05) and LPS (p<0.05) when compared to those in the young group (Fig. 4). Responses to Con A and PWM were significantly lower in the adult group of animals, relative to those in the young group.

### ELISPOT Assay for Detecting Antigen-specific IFN-γ-producing Cells

**Figure 5:**
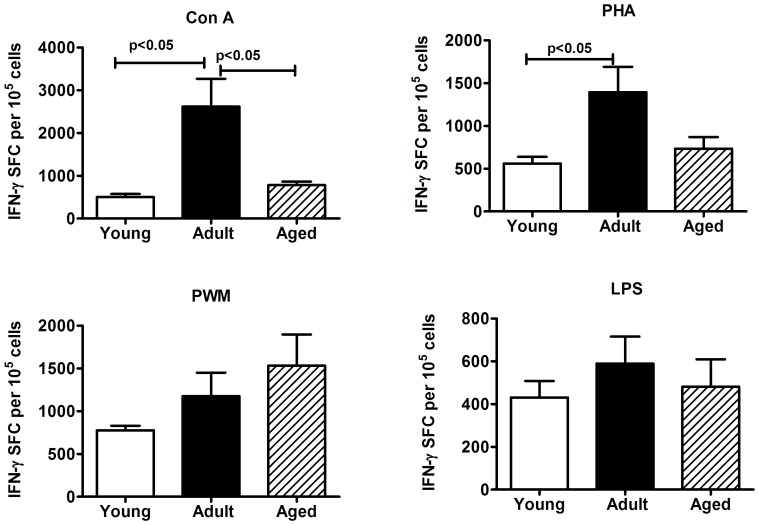
IFN-γ ELISpot response to mitogens. Triplicate wells of the 96-well microtiter plates, pre-coated with IFN-γ antibody, were seeded with 10^5^ PBMC from the monkeys in the three different age groups studied and stimulated with 5 µg of each of the mitogens for 36 h at 37°C followed by washing and staining with biotynylated second IFN-γ antibody. The total number of spot forming cells (SFC) in each of the mitogen-stimulated wells was counted and adjusted to control medium as background. See methods section for experimental details. The results shown are average of two separate experiments and the standard deviation values did not exceed 15% of the mean value. P values were considered at p<0.05.

Aliquots of PBMCs were analyzed for the numbers of cells producing IFN-γ in response to stimulation with Con A, PHA, PWM and LPS by the cytokine ELISPOT assay. As shown in Fig. 5, the adult group of monkeys, relative to those in the young as well as aged groups showed significantly higher numbers of IFN-γ producing cells in response to stimulation with Con A (p<0.05). Similar results were obtained in response to stimulation with PHA, but the difference between adult and aged groups did not reach significance. While no significant differences were observed for the numbers of IFN-γ producing cells in response to stimulation with PWM and LPS in the animals from the three different age groups tested, there was a trend for increasing responses to PWM with age (Fig. 5).

### Plasma Cytokines

**Figure 6:**
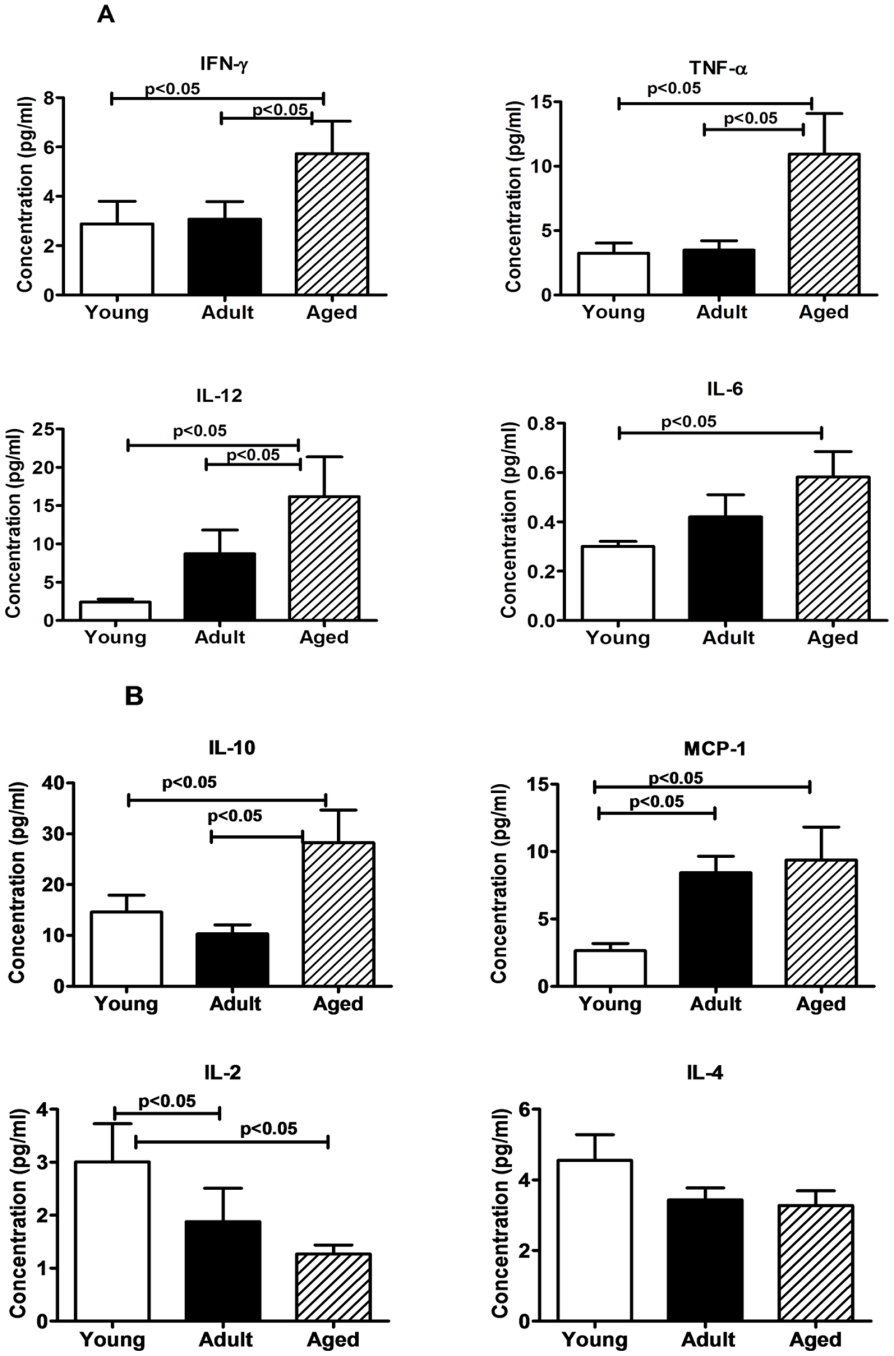
Cytokine Bead array (CBA) analyses of plasma samples. In duplicate wells of the 96-well filter plate 25 µl of plasma was incubated with 25 µl of cytokine coupled beads for overnight at 4°C followed by washing and staining with biotynylated detection antibody. The plates were read on Biorad 200 using Luminex technology and the results are expressed as pg/ml concentration of (**A**) IFN-γ, TNF-α, IL-12, and IL-6; (**B**) IL-10, MCP-1, IL-2, and IL-4. The minimum detectable concentrations in pg/ml for IL-2 (0.7), IL-4 (2.7), IL-6 (0.3), IL-10 (6.2), IL-12(P40) (1.2), IFN-γ (2.2), and TNF-α (2.1) are used for considering positive responses. See methods section for experimental details. The results shown are average of two separate experiments and the standard deviation values did not exceed 15% of the mean value. P values were considered at p<0.05.

We have analyzed plasma samples from the monkeys in the three different age groups for seven different cytokines (IL-2, IL-4, IL-6, IL-10, IL-12, IFN- γ and TNF-α) and the chemokine MCP-1, using the bead array kit, and observed significantly higher levels of IL-6 (p<0.05), IL-10 (p<0.05), IL-12 (p<0.05), IFN-γ (p<0.05), TNF-α p<0.05) and MCP-1 (p<0.05) in the aged monkeys compared to that in both the adult and young animals (Fig. 6 A and B). However, with respect to IL-2 we observed a reverse trend with significantly lower levels in adult and aged population compared to that in the young animals, while IL-4 levels showed a declining trend with age that did not reach statistical significance (Fig. 6 A and B).

## Discussion

Non-Human Primate (NHP) models for studying age-associated effects in humans offer some distinct advantages based on their genetic and biologic similarity to humans. For instance, co-morbidity patterns in aging monkeys closely mirror those seen in humans including the development of age-related diseases such as diabetes, hypertension, pancreatic and neurologic amyloid deposition, and atherosclerosis. The prevalence of these diseases in NHP increases with age and with the consumption of a western diet, as described for humans [26], [27]). Furthermore, NHPs are susceptible to either human pathogens or simian pathogens that bear significant homology to human infectious agents [5]–[7], [22], [28]. While the focus of ageing research in non-human primates has been on the rhesus macaques (Macaca mulatta), an increased interest exists in the use of squirrel monkeys due to the environmental and infectious disease exposures that are comparable to that of humans [2], [3], [5]–[7], [28]. In the face of the increased interest, the literature is lacking significant contributions from this species with regards to various immune parameters in different age groups.

Data from the present investigation showed that healthy female squirrel monkeys representing three different age groups arbitrarily classified as young, adult and aged exhibit comparable levels of peripheral blood lymphocytes representing the CD3, CD4 and CD8 T cells subsets. This finding is in accordance with the several studies in the literature in humans and other nonhuman primate species that reported stable pools of circulating T lymphocytes subsets during ageing. [8], [29]–[32]. However, in the squirrel monkeys we observed that CD4^+^CD8^+^ double-positive (DP) T cells were significantly reduced (p<0.05) in young population compared to adults and aged. There are sporadic reports describing age-related increase of peripheral DP T cells in humans as well as in animals such as mice, chicken, swine and monkeys [33]–[37], but their function and biological significance are not well understood. Monkeys with a higher percentage of T cells with this phenotype were also observed to exhibit higher percentages of single positive CD4 and CD8 T cells with memory phenotype [33]. In people with human immunodeficiency virus (HIV) or Epstein Barr Virus (EBV) infections, it is observed that the percentage of DP T cells can increase to 20% of all circulating lymphocytes [38]–[40]. Therefore, it is believed that T cells with this phenotype in the periphery participate in the adaptive immune response against infectious pathogens, increasing the repertoire of the T cell antiviral immunity. Our studies also showed a significant decrease (p<0.05) of the CD20+ B cells in adult and aged female squirrel monkeys compared to young. This is in accordance with reports describing age-associated changes in mice and human immune system for a decrease in number of B cells in the periphery [41], [42].

Even though, the numbers of circulating CD4+ or CD8+ T cells subsets did not significantly differ in the three different age groups, we observed significantly higher percentages of central and effector memory subsets of CD8+ T cells in the aged group animals relative to those in the adult and young groups. With respect to the naïve CD8+ T cells, a reverse was true with the young animals showing significantly higher percentages compared to animals in the adult and aged animals. These findings are similar to reports by others [12], [43], including a study on indoor-housed rhesus macaques reported by Cicin-Sain and colleagues [44] comparing mean levels between younger (aged 6–9 years old) and older (aged 18–24 years old) rhesus macaques.

We also analyzed for the expression levels of CD28 and CD95 markers on CD4+ and CD8+ T cells. One major biomarker of T cells in older populations of humans and animals is a decrease in the expression of CD28 and since this is an essential co-stimulatory molecule, its decreasing expression with age could be one underlying cause for the compromised capacity to respond to activation signals in these cells. We observed the expression levels of CD28+ on both CD4+ and CD8+ T cells to be highest in the animals from the young group with a gradual declining trend in the adult and aged groups of animals [45]. In contrast, we observed increasing trend for the expression of the apoptosis-related molecule CD95, on CD4+ and CD8+ T cells in animals from the young to adult to the aged groups. These data suggest that the expression of CD95 on the different subsets of lymphocytes can be considered a good marker for studies of immunosenescence, because it may be predictive of aging, and can partially explain the functional changes in the lymphocytes subsets in elderly [46].

Numerous reports in humans and animals described an inverse relation between age and capacity to mount antigen specific immunity and therefore associated in aged populations with increased susceptibility to infections, failure to respond to vaccines, and chronic inflammation [16], [47]–[49]. This age-related decline in T cell function is believed to be preceded by involution of the thymus gland (cortex involutes much more than the medulla), with dramatic declines in thymic hormone levels seen in animals and humans. In addition, as aging occurs there is an expansion of incompetent memory lymphocytes and decreased proliferative capacity and impaired expression of IL-2 [50]. In further support of this contention, in the present investigation we observed in the aged group of the squirrel monkeys, relative to those in the adult and young groups, decrease in proliferation responses to a variety of mitogens (PHA, ConA, PWM, and LPS), as well as significantly reduced IFN-γ producing cells in response to stimulation with PHA and Con-A. It is however important to note here that the data presented here is obtained using total PBMC for stimulation with the different mitogens, and responses specific to T and B lymphocytes as well as CD4+ and CD8+ subsets of T cells will have to be assessed independent of each other to understand the significance of the contributions of these individual cell types.

Comparison of the plasma concentration of cytokines and chemokines demonstrated that IL-6, IL-10, IL-12, IFN- γ, TNF-α, and MCP-1 levels were significantly higher in aged subjects than young and adult subjects, whereas IL-2 was significantly lower in adult and aged subjects (Fig. 6 B). Our findings are similar to the previous studies in humans where these cytokines levels were elevated in elderly subjects compared to younger subjects [51]–[53]. To our knowledge, this is the first study that has compared profiles of cytokine and chemokine, in young, adults and aged population of squirrel monkeys. Monocytes, a key component of the first line of defense, are among the primary inflammatory cell types recruited to local tissue sites in response to infection or inflammation. The chemonkine, MCP-1 (CCL2), a member of the CC chemokine family, regulates migration of monocytes by promoting their exit from the bone marrow into the circulation to reach the site of inflammation [54], [55]. In our study we found the aged group of squirrel monkeys had significantly higher levels of MCP-1 relative to the young and adult groups, a finding similar to that reported for aged human population [56].

No significant differences were found between the different age groups of the female squirrel monkeys in the present study for RBC, HGB, HCT, MCV, MCH, MCHC, and RDW. This is in contrast to the reports in aging captive rhesus macaque population where a significant decline in MCV and MCH along with significant increase in RBC, Hgb, HCT were reported [57], [58]. We also observed no changes in circulating levels of WBC, lymphocytes, monocytes, neutrophils, eosinophil and basophils between animals in the different age groups, which is similar to that previously reported in rhesus macaque [57], [58].

It is important to point out that due to the limited availability of cross reactive molecular and immunological research reagents resources for squirrel monkeys cells, we could only perform a limited study of the phenotype and function of squirrel monkey cells. Nevertheless, our current study demonstrates that majority of phenotypic and functional aspects of lymphocytes in the whole blood of squirrel monkeys representing different age groups ranging between 3 and 19 years are similar to those reported in aging populations of other nonhuman primate species and also humans. The squirrel monkey, being a Neotropical primate, carries less risk to personnel compared to macaques and other Old World primates. Furthermore, the small size of the squirrel monkey allows much smaller doses of vaccine and therapeutic reagents to be used.

In summary, significant differences exist in immune system functionality between young and older female squirrel monkeys. Specifically, older squirrel monkeys have a pro-inflammatory cytokine profile and do not respond as robustly to mitogens as their young counterparts. Overall, data from this investigation should further support the value of squirrel monkeys as a useful animal model for human studies and the specific differences noted for this species relative to other nonhuman primates and humans should assist researchers for informed analyses of immune parameters with appropriate adjustments for the noted differences. Additional research is needed to determine if these findings from the different age groups of female animals are also seen in male squirrel monkeys and whether the pathogenesis sequela of these findings is comparable to humans.
